# Targeted Quantitative Analysis of Specific Proteins in Cytosolic, Mitochondrial, and Nuclear Fractions Using PRM

**DOI:** 10.1021/acs.jproteome.5c00697

**Published:** 2026-02-25

**Authors:** Alejandra. Delgado-Sequera, Alberto Paradela, Fernando J. Corrales

**Affiliations:** † Functional Proteomics Laboratory, National Center for Biotechnology (CNB-CSIC), Madrid 28049, Spain; ‡ CIBERehd, Institute of Health Carlos III, Madrid 28029, Spain

**Keywords:** PRM, mitochondrial enrichment, liver, subcellular fractionation, target proteomic

## Abstract

Mitochondria play a central role in liver physiology by regulating key metabolic processes. Consequently, mitochondrial dysfunction is a hallmark of multiple liver diseases, including steatosis, steatohepatitis, and liver failure following hepatectomy. Subcellular fractionation is widely used to isolate mitochondria from liver cells or tissue; however, the enrichment and purity of isolated fractions are critical to ensure reliable downstream functional and proteomic analyses. Conventional validation methods, such as immunoblotting of organelle-specific markers, are limited by low throughput, restricted sensitivity, and variability. In this study, we present a targeted proteomics strategy based on parallel reaction monitoring (PRM) to quantitatively assess the enrichment of cytosolic, mitochondrial, and nuclear fractions obtained from liver samples using commercial isolation kits. PRM analyses demonstrated robust and compartment-specific enrichment in both PLC/PRF/5 cells and mouse liver tissue. In PLC/PRF/5 cells, high nuclear/cytosolic enrichment was observed for Prelamin A/C, while mitochondrial markers such as ATPase showed strong mitochondrial/cytosolic ratios. Cytosolic markers consistently displayed enrichment in the cytosolic fraction. Similar trends were observed in mouse liver tissue, confirming applicability across biological systems. Overall, these results highlight PRM as a sensitive, reproducible, and cost-effective alternative to immunodetection approaches for evaluating subcellular fraction purity, supporting high-quality mitochondrial preparations for translational hepatology studies.

## Graphical abstract

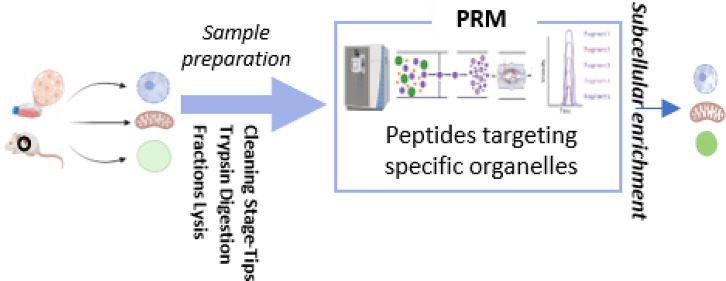

## 1 Introduction

The mitochondrion is the main energy production site of the cell and plays a pivotal role in numerous physiological processes. A deficiency of mitochondrial activity is associated with the pathogenesis of a wide range of diseases^1^ through impairment of energy metabolism, disruption of calcium homeostasis, failures in detoxification systems^2^), and defects in cellular repair pathways.^3^ As mitochondria is a masterpiece governing cell fate, the study of mitochondria has become a central topic in biomedicine. The analysis usually begins with isolation or enrichment procedures^3^ to then measure essential functions such as metabolic activity, respiration, protein import, interactions with other cellular structures, and membrane fusion.^4^

The liver contains one of the highest mitochondrial densities among body organs, reflecting its dependence on oxidative metabolism and mitochondrial-mediated detoxification.^5^ Aberrant mitochondrial function contributes to impaired oxidative phosphorylation, defective fatty acid oxidation, and redox imbalance^6–8^ —key drivers of steatosis and steatohepatitis,^9^ which may progress to cirrhosis and hepatocellular carcinoma.^10–12^ Therefore, the detailed study of isolated mitochondria has significantly extended the knowledge about the molecular pathogenesis of metabolic dysfunction associated fatty liver disease (MAFLD), alcoholic liver disease, and posthepatectomy liver failure.^6,7^ Moreover, isolated mitochondria provide a platform to evaluate the direct impact of drugs, toxins, or metabolic stressors on mitochondrial function, bypassing compensatory cellular responses.^13^ This reductionist model is especially relevant in translational hepatology, where the identification of mitochondrial vulnerabilities and adaptive responses may inform therapeutic strategies or serve as biomarkers of hepatic injury.^14,6,7^ Understanding mitochondrial dynamics and regulation appears then as a relevant issue in understanding liver physiology and pathology. To this end, isolation procedures have been developed which provide mitochondrial preparations whose enrichment degree must be evaluated.

Careful assessment of the enrichment and purity of mitochondrial preparations is essential, as contaminants from other organelles can compromise the accuracy of downstream functional and proteomic analyses^15,16^ The isolated mitochondrial fraction may contain contaminants such as plasma membranes, lysosomes, peroxisomes, or endoplasmic reticulum. These contaminants can interfere with the interpretation of assays measuring calcium transport, oxidative stress or respiration, potentially leading to inaccurate results if the signal originates from nonmitochondrial sources.^4,17^ This consideration is particularly critical in mitochondrial proteomics studies, where contamination with proteins from other cellular compartments can induce misleading functional assignments.^18^

A widely used method to assess mitochondrial enrichment following isolation is Western blotting combined with immunodetection of organelle-specific proteins.^18^ These antibody-based techniques allow for the identification and semiquantitative analysis of mitochondrial markers as well as potential contaminants from other cellular compartments. However, while effective, such methods have inherent limitations that can impact the accuracy and throughput of mitochondrial purity assessments, including the availability of antibodies, cross-reactivity, and the lengthy, multistep nature of the procedure.^15,16^

New technological advances provide alternatives to overcome these challenges using targeted mass spectrometry, specifically Parallel Reaction Monitoring (PRM). PRM enables the simultaneous detection and quantification of a selected panel of protein markers representing various subcellular compartments without relying on affinity reagents.^19^ This approach leverages the unique peptide sequences of target proteins, thus providing a high degree of specificity and sensitivity that surpasses traditional immunodetection methods.^20,21^ Moreover, PRM offers significantly higher reproducibility and throughput, allowing rapid analysis of multiple samples with consistent quantitation.^19,22,23^

Here we describe a method to assess the enrichment degree of cytosolic, mitochondrial, and nuclear fractions which were isolated using different commercial isolation kits from liver cells and liver tissue. Given the focus of our research, we selected hepatic tissue and cell-based systems as the primary objects of study. Our aim was to develop and evaluate a rapid and straightforward PRM-based method to assess mitochondrial, nuclear, and cytoplasmic enrichment. We did not seek to evaluate the efficiency of commercial fractionation kits, nor to perform comparisons with traditional protein quantification methods. Our findings demonstrate the robustness and versatility of PRM for assessing mitochondrial enrichment, making it a cost- and time-effective tool to ensure the quality of mitochondrial preparations, a critical factor for a downstream functional and proteomic analyses.

## 2 Methods

### 2.1 Biological Samples

FVB wild-type mice were purchased from Charles River. Two grams of liver specimens were obtained from 12 to 15-week-old mice (*n* = 3 biological replicates). Tissue samples were frozen in liquid nitrogen immediately after extraction and stored at −80 °C until use.

PLC/PRF/5 cells (*n* = 3 biological replicates) were cultured in DMEM supplemented with 10% FBS, 1% Glutamine and 1% penicillin-streptomycin at 37 °C in a 5% CO_2_. Cells were grown to 80% confluence and collected by scraping.After centrifugation at 1500*g* for 5 min cell pellets were washed thrice with ice cold PBS and the final pellet was stored at −80 °C until use.

### 2.2 Isolation of Subcellular Fractions

Mitochondria and cytosol were isolated from mouse liver using the Mitochondria Isolation Kit for Tissue (Pierce, 89874 USA). Mitochondria and cytosol from PLC/PRF/5 cells (*n* = 3 biological replicates) were obtained using the Mitochondria Isolation Kit for Cultured Cells (Pierce), according to the manufacturer’s instructions. Nuclear fractions from liver tissue and PLC/PRF/5 cells were isolated using the Nuclei PURE Prep Nuclei Isolation Kit (Sigma, NUC-201, USA), following the manufacturer’s protocol (Figure 1A).

**Figure 1:**
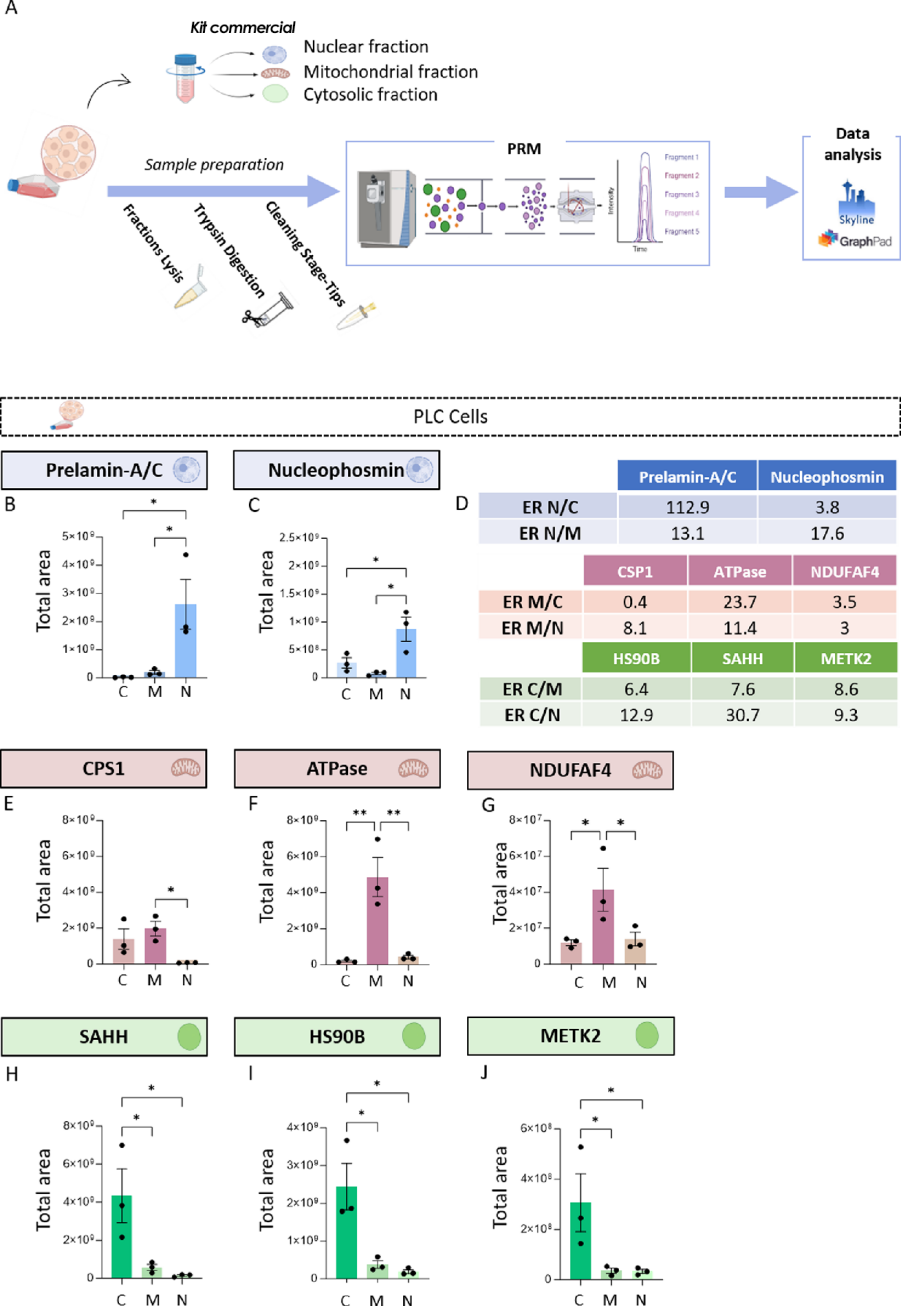
Monitoring subcellular fractionation by PRM in PLCs. (A) Representation of the analytical pipeline in PLCs. Starting from PLCs, cytosolic, mitochondrial, and nuclear fractions were extracted. Then, subcellular proteomes were analyzed using a PRM protocol to monitor selected subcellular fraction marker proteins. The analysis involved precursor ion selection in Q1, fragmentation in Q2, and final detection in the Orbitrap analyzer. Data processing and quantification were carried out using *Skyline* software, and statistical analysis was performed using GraphPad Prism. (B) Graphical representation of the total area for prelamin A/C in subcellular fractions from cells. The total area corresponds to the sum of the fragment ion peak intensities across the chromatographic elution profile and serves as a proxy for the relative abundance of the peptide. The abbreviations C, M, and N correspond to cytosolic, mitochondrial, and nuclear fractions, respectively. (C) Graphical representation of the total area for Nucleophosmin. (D) ER indicates enrichment ratios between nuclear (N), cytosolic (C), and mitochondrial (M) fractions. Nuclear (blue), mitochondrial (pink), and cytosolic (green) marker proteins show preferential enrichment in their corresponding fractions, supporting effective subcellular fractionation. (E) Graphical representation of the total area for CPS1. (F) Graphical representation of the total area for ATPase. (G) Graphical representation of the total area for NDUFAF4. (H) Graphical representation of the total area for SAHH. (I) Graphical representation of the total area for HS90B. (J) Graphical representation of the total area for METK2. Individual data points represent biological replicates. Data are presented as mean ± SEM *p < 0.05; ** *p* < 0.01; *p < 0.0001.

### 2.3 Sample Preparation

Isolated mitochondria and nuclei were lysed in lysis buffer [7 M urea, 2 M thiourea, 4% CHAPS (3-[(3-cholamidopropyl) dimethylammonio]-1-propanesulfonate), 40 mM dithiothreitol (DTT) at pH 7.7] for 30 min at 4 °C, followed by two rounds of sonication for 20 s each, with a 20-s pause between cycles. Lysates were centrifuged at 17,000 × *g* for 20 min at 4 °C, and protein concentration was determined in the supernatant using the Pierce 660 nm Protein Assay (Thermo Fisher Scientific). Protein processing (30 μg) was done in micro S-Trap columns (Protifi). Briefly, reduction and alkylation of Cys sulfhydryl groups were performed by incubation with tris (2-carboxyethyl) phosphine (TCEP, 5 mM) and chloroacetamide (CCA, 10 mM), for 1 h at 37 °C. Protein digestion was performed with trypsin (90057, Thermo Fisher Scientific) at a 1:15 ratio (μg trypsin: μg protein) o/n at 37 °C. The resulting peptide mixture was cleaned with stage -Tips (Empore Octadecyl C18 extraction disks, SUPELCO) and the concentration was then determined using a Qubit fluorometric assay (Invitrogen).

### 2.4 Definition of the PRM Method

In order to assess the enrichment degree of the mitochondrial, cytosolic and nuclear fractions, six reporter proteins were selected whose subcellular location has been reported to be restricted to specific compartments: NPM (UniProtKB: P06748) for the nuclear fraction, HSP90AB1 (UniProtKB: P08238),AHCY (UniProtKB: P23526)­and METK2 (UniProtKB: P31153) for the cytosolic fraction, and CPS1 (UniProtKB: P31327), ATP5F1 (UniProtKB: P25705) and NDUFAF4 (UniProtKB: Q9P032) for the mitochondrial fraction (Table 1)*.*

**Table 1:** PRM Transitions Monitored to Estimate the Enrichment of Purified Cytosolic, Mitochondrial, and Nuclear Cellular Fractions

protein	gene	code human/mouse	peptide	mass (m/z)	CS [z]	transitions
prelamin-A/C	LMNA	P02545/P48678	EGDLIAAQAR	522.278	2	y7+, y6+, y5+, y4+, b3+
			LADALQELR	514.790	2	y8+, y7+, y6+, y5+, b2+
nuclephosmin	NPM	P06748/P13084	VDNDENEHQLSLR	784.869	2	y6+, y5+, y4+, y3+, y1+
ATP synthase subunit alpha, mitochondrial	ATP5F1A	P25705/Q03265	ILGADTSVDLEETGR	788.397	2	y13+, y11+, y10+, y9+, y7+
			VLSIGDGIAR	500.793	2	y8+, y7+, y6+, y4+
			TGAIVDVPVGEELLGR	812.949	2	y12+, y11+, y10+, y9+, b3+
carbamoyl-phosphate synthase [ammonia], mitochondrial	CPS1	P31327/Q8C196	IALGIPLPEIK	582.373	2	y6+, y4+, y2+, b2+, b4+
NDUFAF4	NDUFAF4	Q9P032/Q9D6J6	EQISLYPEVK	603.324	2	y7+, y5+, y4+, y2+, b2+
			IMQEYQLEQK	437.220	3	y4+, y3+, y2+
METK2	MAT2A	Q9Y5K3/Q9DBP5	TQVTVQYMQDR	684.832	2	y9+, y8+, y6+, b2+
			YLDEDTIYHLQPSGR	903.936	2	y8+, y7+, y6+, y5+, y4+,
			FVIGGPQGDAGLTGR	722.880	2	y13+, y12+, y8+, y6+, b2+
heat shock protein HSP 90-beta	HSP90AB1	P08238/P11499	YHTSQSGDEMTSLSEYVSR	726.320	3	y9+, y8+, y7+, y6+, b9+
			HLEINPDHPIVETLR	594.989	3	y7+, y5+, b2+, b5+, b7+
adenosylhomocysteinase	AHCY	P23526/Q8BWF0	VPAINVNDSVTK	628.846	2	y10+, y9+, y8+, b2+, b3+
			IILLAEGR	442.782	2	y6+, y5+, y4+, y2+, b2+

The selection of proteotypic peptides for inclusion in the PRM assay was based on a combination of **in-house shotgun proteomics data** (unpublished results) and publicly available information from **UniProt** (https://www.uniprot.org) and **PeptideAtlas** (http://www.peptideatlas.org). In-house data were generated through shotgun proteomic analysis of **PLC/PRF/5 cell samples**, and only the **most abundant proteins** detected in these experiments were considered as candidates for PRM peptide selection.

The selection of proteotypic peptides followed several stringent criteria to ensure reliability and detectability:

1. **Concordance with experimental and public data**: Peptides had to be detected both in our in-house shotgun results and reported in public databases, ensuring consistency and reproducibility.
2. **Peptide length**: Only peptides between **8 and 25 amino acid residues** were considered, optimizing for effective fragmentation and MS detectability.
3. **Cleavage specificity**: Peptides with **missed enzymatic cleavages** were excluded to reduce variability in digestion efficiency and improve quantification accuracy.
4. **Avoidance of chemically labile residues**: Peptides containing **methionine (Met), tryptophan (Trp), or other residues prone to modification** either in the cellular context or during sample preparation and analysis were avoided, whenever alternative suitable peptides were available.

This strategy ensured that the selected proteotypic peptides were both experimentally observable and robust for quantitative PRM analysis, minimizing potential sources of variability while maximizing confidence in peptide detection.

### 2.5 nLC-MS/MS Analysis

Tryptic peptides were resuspended at 200 ng/ul, according to QUBIT quantification (Thermofisher Scientific). Five μL of each sample (equivalent to 1 μg) were loaded online on a C18 PepMap 300 μm I.D. 0.3 × 5 mm trapping column (5 μm, 100 Å, Thermo Scientific) and analyzed by LC-ESI MSMS using a Thermo Ultimate 3000 RSLC nanoUPLC coupled to a Thermo Orbitrap Exploris OE240 mass spectrometer. Peptides were separated on a 2 μm particle size, 15 cm × 75 μm ID column, with a flow rate of 300 nL/min and a 60 min long gradient. The liquid chromatographic system was coupled via a nanospray source to the mass spectrometer. The mass-spec method used worked in PRM (parallel reaction monitoring) mode. Experimental settings for PRM analysis initially were set up with the help of Skyline v.24.1 software (MacLean et al.^53^).

### 2.6 Data Processing

Selection and extraction of the signals corresponding to each selected transition (Table 1), was carried out with the Skyline v24.1 software. All results were manually curated and all interferences excluded. Transition areas were summed and presented at the peptide level. Statistical analyses were performed using GraphPad Prism software (GraphPad Software, USA). Statistical differences were assessed using one-way ANOVA, and results were considered significant at a *p*-value <0.05.

## 3 Results and Discussion

### 3.1 Selection of Cell Compartment-Specific Proteins

Eukaryotic cells are organized into membrane-bound compartments with specialized functions,^24^ which coordinate to support the overall cellular machinery. The functional specificity of each compartment is determined by a distinct and dynamic protein landscape, enabling intercommunication between subcellular structures. Understanding the dynamic proteome of organelles is crucial to deciphering their roles in both physiological and pathological contexts. Over the past decades, a variety of protocols have been developed to facilitate such analyses, including modern standardized approaches-reproducible, validated, and widely accepted methods for analyzing subcellular proteomes, in contrast to older, less consistent techniques.^25–27^ Critical steps in the characterization of cellular organelles are their isolation and the evaluation of enrichment procedures. Traditionally, this has been achieved using antibodies targeting organelle-specific proteins, serving as markers to estimate preparation purity. However, the limitations of antibodies go beyond being mere ‘bottlenecks’ for characterization; they can actually hamper accurate determination in subcellular fraction quality control.To address these limitations, we propose a mass spectrometry-based approach employing PRM to detect a defined set of compartment-specific proteins. This targeted strategy offers a robust and antibody-independent alternative for the assessment of organelle enrichment and integrity. Compared to SRM, PRM provides higher specificity, greater selectivity (reduced interferences), improved confidence in identification (based on a full high resolution spectrum) and higher flexibility (easier to expand target lists).^28^

The selection of the marker proteins was based on the current evidence supporting their subcellular distribution, as it has been compiled in several knowledge base resources. UniProt stands out as a comprehensive, manually curated database offering detailed annotations based on experimental evidence and computational predictions.^29^ It provides specific insights into protein compartments such as the mitochondrial matrix, inner membrane, cytosol, or nuclear lamina, making it a reliable primary source. Complementing UniProt, the Human Protein Atlas (HPA) adds a valuable layer of data through immunofluorescence imaging, which visually confirms protein distribution within different organelles across human cell lines.^30^ This imaging-based approach allows researchers to observe protein localization in situ, enhancing confidence in compartment assignment. GeneCards (https://www.genecards.org/) further integrates information from multiple databases, including UniProt and HPA, offering a synthesized overview of protein properties and subcellular localization.^31^ Although less detailed, GeneCards is a convenient starting point for researchers seeking quick summaries and links to primary data. Additionally, the Compartments database (https://compartments.jensenlab.org/Search) aggregates evidence from various experimental and computational sources and provides confidence scores for each predicted localization.^32^ Its interactive visualization tools facilitate an intuitive understanding of protein distribution across cellular compartments, which is particularly helpful when assessing proteins with multiple localizations or ambiguous assignments. NCBI Gene, while generally less detailed in localization data, can serve as a complementary resource, often linking out to more specialized databases.^33^ Together, these resources form a robust framework for accurately characterizing protein subcellular localization. Using multiple sources not only enhances the reliability of localization assignments but also provides diverse perspectives—ranging from high-resolution curated annotations to imaging-based validation and integrated confidence scoring—that are crucial for interpreting experimental proteomic data and understanding protein function within cellular contexts.

To accurately evaluate the purity and enrichment of each subcellular fraction, six well-characterized protein markers were selected for mitochondria, cytosol, and nucleus, respectively. For mitochondria, CPS1, ATP5F1 and NDUFAF4 were chosen. CPS1 is an enzyme primarily located in the mitochondrial matrix, where it catalyzes the first step of the urea cycle, facilitating ammonia detoxification.^34^ ATP5F1 is a key component of the ATP synthase complex in the mitochondrial inner membrane, which is essential for ATP synthesis during oxidative phosphorylation.^35^ NDUFAF4 is a mitochondrial protein that functions as an essential assembly factor for complex I of the oxidative phosphorylation system. It is localized to the mitochondrial inner membrane/matrix side and plays a critical role in the early stages of complex I biogenesis, contributing to the stability and proper incorporation of core subunits required for functional electron transport.^36,37^ For the cytosolic fraction, HSP90AB1, AHCY and METK2 were selected. HSP90AB1 (Heat Shock Protein 90 Beta Family Member 1) is an abundant cytosolic chaperone that assists in protein folding and stabilization, helping to maintain cellular homeostasis under stress conditions.^38^ AHCY (S-Adenosylhomocysteine Hydrolase) is a cytosolic enzyme involved in methylation metabolism by hydrolyzing S-adenosylhomocysteine, a process critical for epigenetic and metabolic regulation^39^ METK2 catalyzes SAM synthesis, supporting methylation, tumor growth, and metabolic adaptation; its inhibition impairs proliferation in MTAP-deficient cancers and tissue regeneration.^40,41^ Finally, for the nuclear fraction, Prelamin A/C and NPM were used as markers. Prelamin A/C is a structural protein of the nuclear lamina located at the nuclear envelope, providing mechanical support and regulating chromatin organization.^42^ NPM (Nucleophosmin) is primarily localized in the nucleolus and plays an important role in ribosome biogenesis and cell cycle regulation.^43^ These markers are proposed as reliable indicators of compartment-specific enrichment and potential cross-contamination, which is essential for downstream functional assays and proteomic analyses (Table 1).

### 3.2 Evaluation of the PRM Method in Subcellular Fractions from PLC/PRF/5 Cells

To monitor the enrichment of the nuclear fraction, the levels of prelamin A/C and nucleophosmin were measured as nuclear markers. Both proteins were significantly more abundant in the nuclear fraction compared to the cytosolic fraction (*p* = 0.0273 and *p* = 0.04636, respectively; one-way ANOVA followed by Tukey’s post hoc test) (Figure 1B,C; Table S1), as well as when compared to the mitochondrial fraction (*p* = 0.0364 and *p* = 0.0148, respectively;) (Figure 1B,C;Table S1). The mitochondrial fraction purity was evaluated by monitoring the proteins CPS1, ATPase and NDUFAF4. CPS1 levels were not found to be significantly different in the mitochondrial fraction compared to the cytoplasmic fraction (*p* = 0.5967) (Figure 1E; Table S 1), whereas a similar trend was observed when compared to the nuclear fraction (*p* = 0.0357) (Figure 1E; Table S 1) Likewise, ATPase showed significantly higher levels in the mitochondrial fraction compared to both the cytosolic and nuclear fractions (*p* = 0.0047 and *p* = 0.0060, respectively) (Figure 1F; Table S 1). On the other hand, we observed a statistically significant increase of NDUFAF4 protein in the mitochondrial fraction compared to both the cytoplasmic and nuclear fractions (*p* = 0.0282; *p* = 0.0365) (Figure 1G; Table S 1).

Finally, the enrichment of the cytosolic fraction was evaluated by monitoring and quantifying the levels of SAHH and HS90B and METK2. The present study has revealed that all of the proteins under investigation were found to be significantly more abundant in the cytosolic fraction than in the mitochondrial fraction (SAHH: *p* = 0.0413; HS90B: *p* = 0.0159; METK2: *p* = 0.0287) (Figure 1H–J; Table S 1). Furthermore, in comparison with the nuclear fraction, the levels of all proteins were found to be significantly elevated (SAHH: *p* = 0.0267; HS90B: *p* = 0.0106; METK2: *p* = 0.0276) (Figure 1H–J; Table S 1). Conversely, the enrichment ratios (ER) in the nuclear fraction were found to be substantially elevated, particularly in comparison to the mitochondrial fraction, exhibiting levels of enrichment that exceeded 10-fold. In comparison with the cytoplasmic fraction, the ER demonstrated elevated levels, albeit with greater variability. This variability was over 100-fold for Prelamin-A/C and approximately 4-fold for nucleophosmin (Figure 1D).

The variability of the proteins was found to be high in general, as indicated by the CV% (Table S2). In order to verify that this variability is predominantly attributable to discrepancies in the extraction of these fractions within their biological replicates, as opposed to inherent flaws in the PRM method itself, we conducted technical replicates of certain proteins as a control. For the nuclear fraction, Prelamin-A/C demonstrated coefficients of variation below 20% across all fractions: cytosol (C) 16.7%, mitochondrial (M) 7.3%, nuclear (N) 5.6%, and total proteome (PT) 7.2% (Figure S1F). This finding suggests that the PRM method is both efficient and precise, and that nuclear isolation is of high quality, as evidenced by the significant elevation in Prelamin-A/C levels relative to the total proteome (*p* < 0.001) (Figure S1A).

For nucleophosmin, the CVs were 10.5% for the cytosolic fraction, 26.6% for the mitochondrial fraction, 15.5% for the nuclear fraction, and 27.3% for the total proteome (Figure S1F). These values were slightly higher than for Prelamin-A/C, but still sufficiently low to consider the method reliable. With regard to the process of nuclear enrichment, the efficacy of the method is evidenced by its superiority in comparison to the total proteome. This is evidenced by a significant elevation in the levels of nucleophosmin in the nuclear fraction (*p* < 0.001) (Figure S1B).

For the mitochondrial fraction, we observed very low variability for CPS1 across subcellular fractions: cytosol 0.3%, mitochondrial 14.1%, nuclear 8.8%, and total proteome 28.7% (Figure S1F). However, ATPase exhibited considerably higher CVs, with values exceeding 50% in the cytosolic, mitochondrial, and nuclear fractions, and 14.9% in the total proteome. This phenomenon may be attributable to the presence of contaminants within the mitochondrial fraction, as indicated by the commercial isolation kit used (*Thermo Fisher, CAT: 89874/89801*). The kit provides a warning that the crude mitochondrial fraction may contain lysosomal or peroxisomal contaminants. Finally, for HSP90B, the CVs were below 15% for all subcellular fractions: cytosol 2.2%, mitochondrial 13%, nuclear 5.8%, and total proteome 12.2% (Figure S1F). With regard to cytoplasmic enrichment, it can be considered effective because HSP90B levels in the cytoplasmic fraction were significantly higher compared to the total proteome (*p* < 0.001) (Figure S1E).

Taking all these results together, we can conclude that the PRM-based assay confirms the efficiency and specificity of the subcellular fractionation protocol used in this study. Marker protein analysis demonstrated robust enrichment of nuclear, cytosolic, and mitochondrial fractions with minimal cross-contamination. The nuclear fraction showed significantly higher levels of prelamin A/C and nucleophosmin, supporting its purity and structural integrity.^44,45^ Mitochondrial enrichment was supportedby the elevated abundance of CPS1, ATPase and NDUFAF4, confirming successful isolation of functionally intact mitochondria, essential for studies on mitochondrial dysfunction and metabolism.^46,47^ Similarly, the cytosolic fraction exhibited strong enrichment of SAHH HS90B and METK2, indicating effective separation from nuclear and mitochondrial components.^48,49^ Together, these findings establish a solid foundation for downstream proteomic and metabolic analyses within distinct subcellular compartments. These results suggest the reliability of the selected proteins as markers of subcellular compartments, provided the efficiency of the fractionation procedures used in this study. It should be noted that the levels of CPS1 are about 30% of those found in the mitochondrial fraction, which may reflect the normal trafficking or distribution of the protein. Alternatively, it may result from a partial enrichment of the mitochondrial fraction that would contain cytosolic proteins as contaminants, although this observation is not supported by the results obtained for ATPase.

### 3.3 Assessment of Subcellular Fractionation and Protein Enrichment by PRM in Liver Tissue

As in the previous section, the same marker proteins were employed to assess subcellular enrichment. Although prelamin A/C and nucleophosmin displayed higher abundance in the nuclear fraction compared to the cytosolic, these differences were not statistically significant when compared to the cytosolic fraction (*p* = 0.2088 and *p* = 0.4373, respectively; one-way ANOVA followed by Tukey’s post hoc test) (Figure 2B,C; Table S1). Similarly, no significant differences were observed when comparing their levels in the nuclear fraction to those in the mitochondrial fraction (*p* = 0.9011 and *p* = 0.9904, respectively) (Figure 2B,C; Table S1), indicating limited nuclear enrichment under the current fractionation conditions. This observation aligns with previous studies reporting the susceptibility of nuclear fractions to cross-contamination, attributed to the structural complexity and spatial proximity of the nucleus to other cellular compartments.^50^

**Figure 2:**
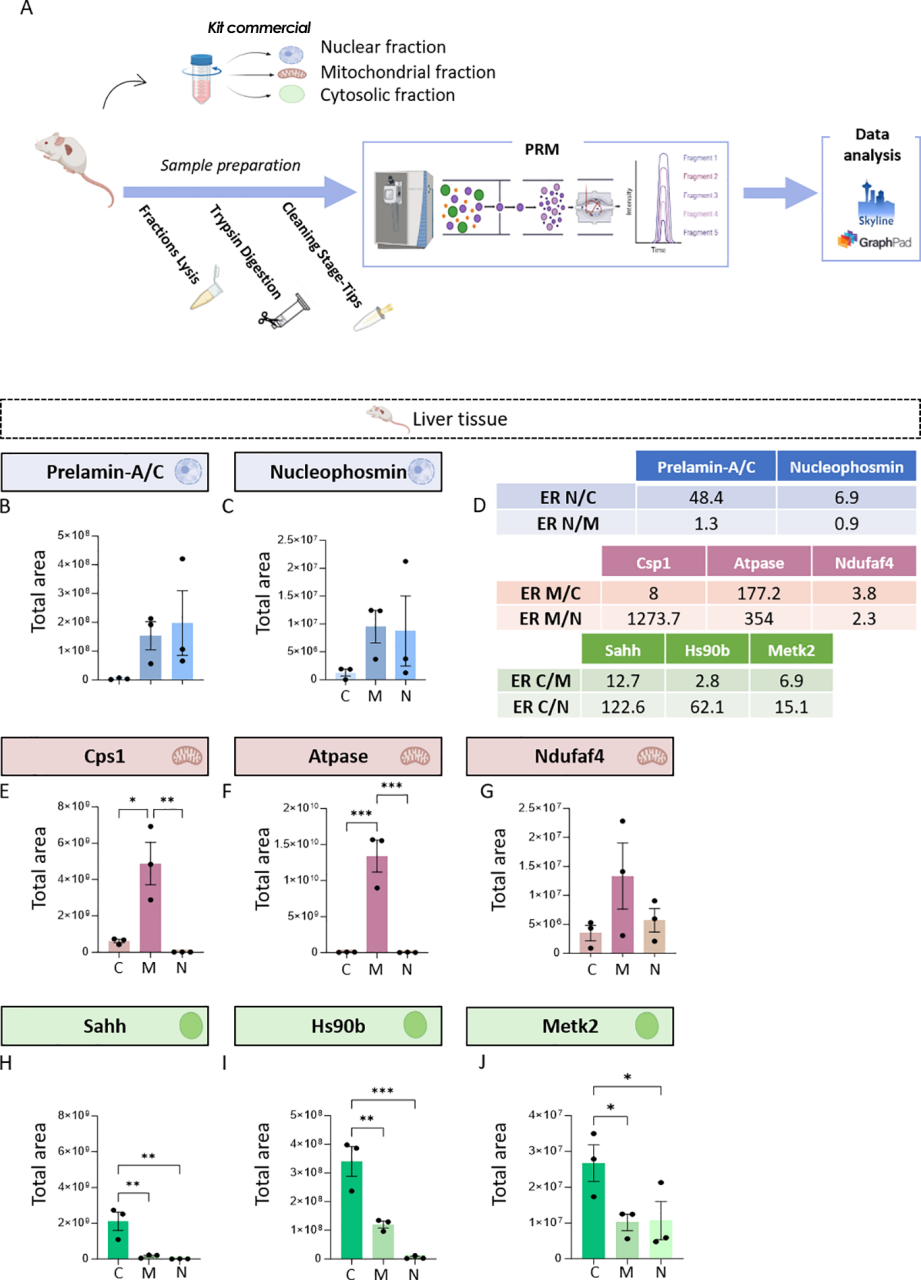
Monitoring subcellular fractionation by PRM in liver. (A) Representation of the analytical pipeline in liver tissues. Cytosolic, mitochondrial, and nuclear fractions were extracted. Then, subcellular proteomes were analyzed using a PRM protocol to monitor selected subcellular fraction marker proteins. The analysis involved precursor ion selection in Q1, fragmentation in Q2, and final detection in the Orbitrap analyzer. Data processing and quantification were carried out using *Skyline* software, and statistical analysis was performed using GraphPad Prism. (B) Graphical representation of the total area for prelamin A/C in subcellular fractions from liver. The total area corresponds to the sum of the fragment ion peak intensities across the chromatographic elution profile and serves as a proxy for the relative abundance of the peptide. The abbreviations C, M, and N correspond to cytosolic, mitochondrial, and nuclear fractions, respectively. (C) Graphical representation of the total area for Nucleophosmin. (D) ER indicates enrichment ratios between nuclear (N), cytosolic (C), and mitochondrial (M) fractions. Nuclear (blue), mitochondrial (pink), and cytosolic (green) marker proteins show preferential enrichment in their corresponding fractions, supporting effective subcellular fractionation. (E) Graphical representation of the total area for CPS1. (F) Graphical representation of the total area for ATPase. (G) Graphical representation of the total area for NDUFAF4. (H) Graphical representation of the total area for SAHH. (I) Graphical representation of the total area for HS90B. (J) Graphical representation of the total area for METK2. Individual data points represents biological replicates. Data are presented as mean ± SEM *p < 0.05; ** *p* < 0.01; *p < 0.0001.

However, Cps1 and Atpase were significantly more abundant in the mitochondrial fraction compared to the nuclear (*p* = 0.0053 and *p* = 0.0008, respectively) (Figure 2E,F; Table S1) and cytosolic fractions (*p* = 0.0101 and *p* = 0.0008, respectively) (Figure 2E,F; Table S1), suggesting the successful enrichment of mitochondrial component and the reliability of the selected markers, paralleling the results from cultured cells experiments. However, a notable discrepancy compared to the cellular model is observed for NDUFAF4. No statistically significant differences were detected between subcellular fractions for this protein, either between the mitochondrial and cytosolic fractions (*p* = 0.1006) (Figure 2G; Table S1) or between the mitochondrial and nuclear fractions (*p* = 0.1829) (Figure 2G; Table S1).

This lack of significance is primarily attributable to the high dispersion of intensity values. Indeed, the coefficients of variation for Ndufaf4 exceeded 60% across all fractions, reaching 66.3% in the cytosolic fraction, 74.3% in the mitochondrial fraction, and 61.2% in the nuclear fraction (Table S2). Conversely, in the cellular approach, the coefficients of variation remained below 50% in all cases (Table S2). Given that the coefficients of variation for technical replicates were within acceptable ranges in most cases, thereby supporting the proper performance of the PRM methodology, the lack of statistical significance is attributed to the high variability among biological replicates. This variability is likely associated with cross-contamination inherent to the commercial isolation kit used, which limits the ability to detect significant differences for Ndufaf4 under these conditions.

On the other hand, Sahh, Hs90b and Metk2 were significantly more abundant in the cytosolic fraction relative to both the mitochondrial (*p* = 0.0088; *p* = 0.0056 and *p* = 0.0397, respectively; One-way ANOVA followed by Tukey’s post hoc test) (Figure 2H–J; Table S1) and nuclear fractions (*p* = 0.0061; *p* = 0.0006 and *p* = 0.044, respectively) as determined by one-way ANOVA with Tukey’s post hoc test (Figure 2H–J; Table S1). These findings confirm our observations in cultured cells and support the selected proteins as checkpoints to evaluate the enrichment of the cytosolic compartment.

One of the limitations of this study is that the PRM-based workflow has been validated solely in mouse liver and PLC/PRF/5 hepatoma cells, which limits its broader biological applicability. Recent studies have indicated significant tissue-specific variability in mitochondrial gene expression and protein composition.^51^ For instance, differential expression of nuclear-encoded mitochondrial genes has been observed across metabolically diverse tissues in buffalo, affecting OXPHOS complex I, ATP synthase subunits, and enzymes involved in amino acid metabolism.^52^ These findings suggest that mitochondrial protein profiles are meticulously adapted to the energetic and metabolic demands of each tissue, indicating that the current PRM marker panel may necessitate refinement or revalidation for tissues with distinct metabolic functions, such as cardiac, skeletal muscle, or neuronal mitochondria. The incorporation of such tissue-specific considerations would enhance the generalizability and applicability of the PRM workflow.

Another consideration relates to the technical accessibility of PRM itself. Although PRM provides high multiplexing capacity, quantitative accuracy, and efficiency in assessing fraction enrichment, its implementation requires access to high-resolution, accurate-mass (HRAM) mass spectrometers and expertise in targeted proteomics data analysis using software such as Skyline.^53,54^ This requirement may limit its immediate accessibility for general biology laboratories. In contrast, traditional immunoblotting, despite lower throughput and semiquantitative nature, is widely used due to its simplicity and broad availability of antibodies.^55^ Therefore, PRM should be considered a complementary, highly robust strategy for laboratories equipped with mass spectrometry infrastructure, rather than a universal replacement for immunoblotting.^56^

A further limitation of this study pertains to the paucity of information pertaining to the functional annotation of subcellular localization in databases such as UniProt and Protein Atlas, which do not always concur. For instance, nucleophosmin is reported by UniProt to be localized to both the nucleus and cytoplasm,^57,58^ whereas Protein Atlas restricts its localization to the nucleus only. CPS1 is listed in UniProt as being associated with the mitochondria, the nucleus, and the membrane,^59^ while Protein Atlas identifies it only in the nucleolus. The UniProt database characterizes ATPase as being associated with both the mitochondrial and plasma membranes,^60,61^ yet Protein Atlas reports exclusive mitochondrial localization. NDUFAF4 is found in mitochondria according to UniProt, but Protein Atlas indicates presence in mitochondria, nucleoplasm, and cytosol. HSP90 shows even greater dispersion: Protein Atlas localizes it to the plasma membrane and cytosol, whereas UniProt reports cytoplasm, melanosome, nucleus, secreted, and cell membrane based on various studies.^62–65^ Similarly, discrepancies exist for SAHH and METK2. UniProt lists SAHH in cytoplasm, melanosome, nucleus, and ER,^66,36^ whereas Protein Atlas reports only cytosol. METK2 is cytosolic in UniProt but both cytosol and nucleus in Protein Atlas. These inconsistencies highlight the limitations of these databases, stemming from differences in annotation methods, heterogeneity of published studies, and variability in cellular contexts analyzed.

Moreover, differences in marker abundance across fractions were interpreted primarily as a reflection of fractionation quality. Physiological states of the tissue or cells—such as stress responses, altered intracellular trafficking, or cell-cycle effects—can influence marker localization in addition of technical fractionation^67^; therefore, some variations may stem from biological conditions, particularly in tissue contexts. Thus, proteins may be detected in subcellular fractions that do not correspond to their canonical localization due to inconsistent database annotations or inherent cross-contamination during fractionation.

Overall, the PRM-based measurement of the selected protein panel has proven to be an efficient strategy for estimating the enrichment level of cytosolic, mitochondrial, and nuclear fractions. The dependence of PRM, SRM, and MRM on specialized instrumentation and user proficiency should be considered to provide a balanced discussion of their advantages and limitations.^68^ Our results highlight not only the reliability of this method but also its value in assessing both the performance of the fractionation protocol and the impact of the biological specimens used. Taken together, this approach contributes to determining the suitability of the sample for downstream proteomic analyses, ensuring precise compartment-specific protein profiling, as previously highlighted in studies focused on mitochondrial function and subcellular proteomics.^69,70^ Moreover, this PRM-based workflow shows promise for application to other tissue types with only minor adjustments, demonstrating its broader adaptability and utility.

## Supplementary Material



## Data Availability

Data are available in the PRIDE repository under accession number PXD067737.

## Notes

PRM-based fraction purity analysis (Figure S1); diagram of the exclusion criteria for selected proteins (Figure S2); relative abundances and statistical analysis across subcellular fractions (Table S1); summary of descriptive statistics (Table S2); and descriptive statistical parameters for technical replicates (Table S3) (PDF)

## Abbreviations

AHCY: S-AdenosilhomocisteIi hidrolasa
ATP5F1: ATP synthase complex
C: cytosolic fraction
CCA: chloroacetamide
CHAPS: (3-[(3-cholamidopropyl) dimethylammonio]-1-propanesulfonate)
DTT: dithiothreitol
HPA: human protein atlas
HSP90AB1: heat shock protein 90 beta family member 1
M: mitochondrial fraction
MAFLD: molecular pathogenesis of metabolic dysfunction associated fatty liver disease
N: nuclear fraction
NPM: nucleophosmin
PRM: parallel reaction monitoring
TCEP: tris (2-carboxyethyl) phosphine
