# Supplementary material for: Targeted Quantitative Analysis of Specific Proteins in Cytosolic, Mitochondrial, and Nuclear Fractions Using PRM
Source: J Proteome Res. 2026 Feb 25;25(4):2183–92. doi: 10.1021/acs.jproteome.5c00697 (PMC13054855; doi:10.1021/acs.jproteome.5c00697)
Supplement: Supplementary file 1 [file pr5c00697_si_001.pdf]

Supplementary information

Targeted quantitative analysis of specific proteins in cytosolic, mitochondrial, and nuclear fractions using PRM

Alejandra. Delgado-Sequera[1], Alberto Paradela[1], Fernando J. Corrales [1,2]*

[1]Functional Proteomics Laboratory, National Center for Biotechnology (CNB-CSIC), Madrid, 28049, Spain.[2]CIBERehd, Institute of Health Carlos III, Madrid, 28029, Spain.

Corresponding Author's email address*: fcorrales@cnb.csic.es*

Table of Contents

1. Figure S1: PRM-based fraction purity analysis
2. Figure S2: Diagram of the exclusion criteria for selected proteins
3. Table S1: Relative abundances and statistical analysis across subcellular fractions
4. Table S2: Summary of descriptive statistics
5. Table S3: Descriptive statistical parameters for technical replicates

11.1 figure S1. **This figure summarizes the relative abundance and variability of selected proteins across subcellular fractions.**

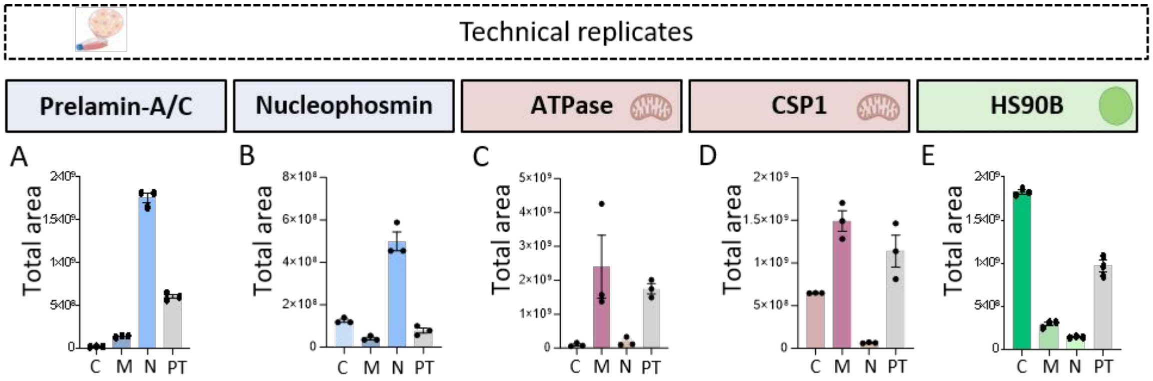

F

CV%	*Cytosol*	*Mitochondrial*	*Nuclear*	*Proteome total*
Prelamin-A/C	16.7	7.3	5.6	7.2
Nucleophosmin	10.5	26.6	15.5	27.3
ATPase	54.8	67.0	72.5	14.9
CSP1	0.3	14.1	8.8	28.7
HS90B	2.2	13	5.8	12.2

Figure S1. **PRM-based assessment of subcellular fraction purity using representative marker proteins.** Bar plots show the relative abundance of (A) Prelamin-A/C, (B) Nucleophosmin, (C) ATPase, (D) CSP1, and (E) HSP90B across cytosolic (green), mitochondrial (pink), nuclear (blue), and total proteome fractions. The abbreviations C, M, N and PT correspond to cytosolic, mitochondrial, nuclear fractions and proteome total, respectively. Individual data points represents technical replicates. The table below summarizes the coefficient of variation (CV%) for each protein in each fraction, providing an estimate of intra-fraction variability. Proteins are color-coded according to their expected predominant subcellular localization.

11.2 Figure S2. **Diagram of the exclusion criteria for selected proteins**

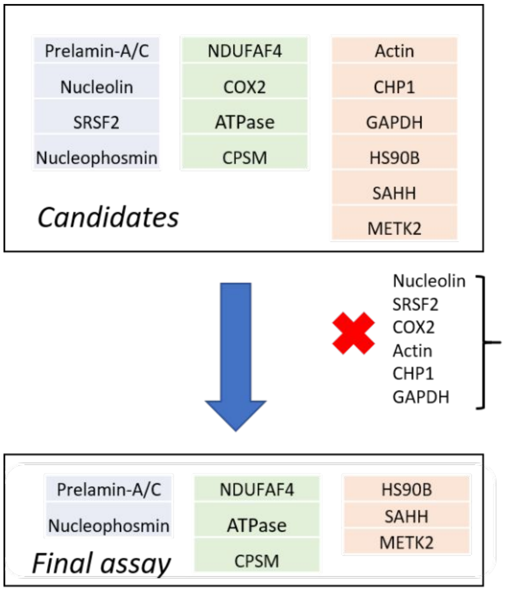

Exclussion criteria:

1. The detected peptides did not meet the predefined selection criteria as defined in section 5.4 of the Materials and Methods.
2. Quality warnings upon manual inspection of peak shape and transition consistency (low signal intensity and limited MS/MS data).

11.3 Table S1. **Relative abundances and statistical analysis across subcellular fractions.**

	PLC/PRF/5							
	**Prelamin-A/C**	**Nucleophosmin**	**CPS1**	**ATPase**	**NDUFAF4**	**HS90B**	**SAHH**	**METKA**
C_1	18466392	11751914	649594176	154924724	9018061	1788563768	2159294336	143978103
C_2	11635317	243295648	1028459136	150842516	12793251	1868264580	3831727232	245463460
C_3	39233640	437563104	2506335232	311054152	14072014	3668018740	6996576640	527924524
M_1	141801012	35176298	1282465536	4256963136	34821733	310562923	281986740	16224452
M_2	123199904	9397108	267193344	3369082368	24930614	246534830	579518976	37868860
M_3	330690208	103343584	195101376	6975700480	64480165	581537448	844411696	52527539
N_1	1811434688	454356032	59075372	331445928	10781811	147081860	59207344	19281609
N_2	1641697216	973127456	76905264	331378720	9723923	131315359	184741442	32241998
N_3	4374997504	1178893248	79969288	619303968	21355624	290248695	178345432	46777037
**Statistical analysis One-way ANOVA (p-values)**								
C vs M	0.97	0.61	0.60	**0.004**	**0.03**	**0.1**	**0.04**	**0.03**
C vs N	**0.03**	**0.05**	0.13	0.97	0.85	**0.1**	**0.03**	**0.03**
M vs N	**0.4**	**0.01**	**0.04**	**0.01**	**0.04**	0.3	0.93	0.98
	Liver tissue							
	**Prelamin-A/C**	**Nucleophosmin**	**CPS1**	**ATPase**	**NDUFAF4**	**HS90B**	**SAHH**	**METK2**
C_1	1312314	0	419631104	49361095	878012	236794800	1094452880	17343659
C_2	7188712	1870768	672593856	81411520	4336501	386527600	2513838848	27876701
C_3	3746799	1902500	729731008	95964286	5299588	398109360	2746079616	34979813
M_1	56473012	3686205	6926065664	8978767232	3061955	96264252	53059655	5620780
M_2	213164588	12362877	4841626624	1.5671E+10	14119953	129327368	217849648	12437175
M_3	191824284	12438886	2880441600	1.5535E+10	22826297	134715400	231102864	12520974
N_1	420719070	21249868	7192829	68235720	2092106	11711988	22875303	5925242

N_2	65877014	1231777	2063053	16751337	5951568	1918158	10052359	4777570
N_3	106457052	3746957	2244933	28519142	9062852	2806653	18884888	21315095
**Statistical analysis One-way ANOVA (p-values)**								
C vs M	0.36	0.38	**0.01**	**0.001**	0.10	**0.005**	**0.009**	**0.04**
C vs N	0.21	0.43	0.81	1.00	0.68	**0.0006**	**0.006**	**0.04**
M vs N	0.90	0.99	**0.005**	**0.001**	0.18	0.09	0.93	0.94

Table S1. **Relative abundances and statistical analysis across subcellular fractions.** The abbreviations C, M, and N correspond to cytosolic, mitochondrial, and nuclear fractions, respectively. For each fraction, three biological replicates were obtained. P-values were calculated using one-way ANOVA for pairwise comparisons between cytosolic vs. mitochondrial, cytosolic vs. nuclear, and mitochondrial vs. nuclear fractions. Data are shown for both the cellular approach (highlighted in yellow) and the tissue approach (highlighted in pink). Differentially significant results are indicated in bold. This presentation allows for direct comparison of protein abundances across subcellular fractions in both experimental contexts

11.4 Table S2. **Summary descriptive statistics**.

	Prelamin-A/C					
	PLC/PRF/5			Liver tissue		
	Cytosol	*Mitochondrial*	*Nuclear*	*Cytosol*	*Mitochondrial*	*Nuclear*
Minimum	11635317	123199904	1641697216	1312314	56473012	65877014
Maximum	39233640	330690208	4374997504	7188712	213164588	420719070
Range	27598323	207490304	2733300288	5876398	156691576	354842056
Mean	23111783	198563708	2609376469	4082608	153820628	197684379
Std. Deviation	14373643	114802262	1531426110	2952556	84978061	194216477
Std. Error of Mean	8298626	66281117	884169277	1704659	49062107	112130935
**CV%**	**62.2**	**57.8**	**58.7**	**72.3**	**55.2**	**98.2**

	Nucleophosmin					
	PLC/PRF/5			Liver tissue		
	Cytosol	*Mitochondrial*	*Nuclear*	*Cytosol*	*Mitochondrial*	*Nuclear*
Minimum	117519140	35176298	454356032	0	3686205	1231777
Maximum	437563104	103343584	1178893248	1902500	12438886	21249868
Range	320043964	68167286	724537216	1902500	8752681	20018091
Mean	266125964	77496987	868792245	1257756	9495989	8742867
Std. Deviation	161238805	36949175	373367006	1089364	5031564	10904143
Std. Error of Mean	93091267	21332616	215563542	628945	2904975	6295510
**CV%**	**60.6**	**47.7**	**43.0**	**86.6**	**53.0**	**124.7**

	CPS1					
	PLC/PRF/5			Liver tissue		
	Cytosol	*Mitochondrial*	*Nuclear*	*Cytosol*	*Mitochondrial*	*Nuclear*
Minimum	649594176	1282465536	59075372	419631104	2880441600	2063053
Maximum	2506335232	2671933440	79969288	729731008	6926065664	7192829

Range	1856741056	1389467904	20893916	310099904	4045624064	5129776
Mean				607318656	4882711296	3833605
Std. Deviation	1394796181	1968470912	71983308	165033713	2023124929	2910594
Std. Error of Mean	981083055	694898430	11283092	95282259	1168051723	1680432
**CV%**	**70.3**	**35.3**	**15.7**	**27.2**	**41.4**	**75.9**

	**ATPase**					
	**PLC/PRF/5**			**Liver tissue**		
	Cytosol	*Mitochondrial*	*Nuclear*	*Cytosol*	*Mitochondrial*	*Nuclear*
Minimum	150842516	3369082368	331378720	49361095	8978767232	16751337
Maximum	311054152	6975700480	619303968	95964286	15671045888	68235720
Range	160211636	3606618112	287925248	46603191	6692278656	51484383
Mean	205607131	4867248661	427376205	75578967	13395023061	37835400
Std. Deviation	91342607	1879164673	166214322	23842784	3825192333	26976933
Std. Error of Mean	52736679	1084936230	95963883	13765638	2208475823	15575140
**CV%**	**44.4**	**38.6**	**38.9**	**31.5**	**28.6**	**71.3**

	NDUFAF4					
	**PLC/PRF/5**			**Liver tissue**		
	Cytosol	*Mitochondrial*	*Nuclear*	*Cytosol*	*Mitochondrial*	*Nuclear*
Minimum	9018061	24930614	9723923	878012	3061955	2092106
Maximum	14072014	64480165	21355624	5299588	22826297	9062852
Range	5053953	39549551	11631701	4421576	19764342	6970746
Mean	11961109	41410837	13953786	3504700	13336068	5702175
Std. Deviation	2627728	20581642	6431966	2325189	9905461	3492058
Std. Error of Mean	1517120	11882816	3713497	1342448	5718921	2016141
**CV%**	**22.0**	**49.7**	**46.1**	**66.3**	**74.3**	**61.2**

	HS90B					
	**PLC/PRF/5**			**Liver tissue**		
	Cytosol	*Mitochondrial*	*Nuclear*	*Cytosol*	*Mitochondrial*	*Nuclear*
Minimum	1788563768	246534830	131315359	236794800	96264252	1918158
Maximum	3668018740	581537448	290248695	398109360	134715400	11711988
Range	1879454972	335002618	158933336	161314560	38451148	9793830
Mean	2441615696	379545067	189548638	340477253	120102340	5478933
Std. Deviation	1062843533	177835853	87564386	89978179	20819427	5416234
Std. Error of Mean	613633000	102673578	50555322	51948926	12020102	3127064
**CV%**	**43.5**	**46.9**	**46.2**	**26.4**	**17.3**	**98.9**

	METK2					
	**PLC/PRF/5**			**Liver tissue**		
	Cytosol	*Mitochondrial*	*Nuclear*	*Cytosol*	*Mitochondrial*	*Nuclear*
Minimum	143978103	16224452	19281609	17343659	5620780	4777570
Maximum	527924524	52527539	46777037	34979813	12520974	21315095
Range	383946421	36303087	27495428	17636154	6900194	16537525
Mean	305788696	35540284	32766881	26733391	10192976	10672636
Std. Deviation	198954930	18263221	13755227	8873491	3959860	9234487
Std. Error of Mean	114866682	10544275	7941584	5123113	2286226	5331533
**CV%**	**65.1**	**51.4**	**42.0**	**33.2**	**38.8**	**86.5**

	SAHH					
	**PLC/PRF/5**			**Liver tissue**		
	Cytosol	*Mitochondrial*	*Nuclear*	*Cytosol*	*Mitochondrial*	*Nuclear*
Minimum	2159294336	281986740	59207344	1094452880	53059655	10052359
Maximum	6996576640	844411696	184741442	2746079616	231102864	22875303
Range	4837282304	562424956	125534098	1651626736	178043209	12822944
Mean	4329199403	568639137	140764739	2118123781	167337389	17270850
Std. Deviation	2456712019	281370283	70703138	894097606	99189023	6562074
Std. Error of Mean	1418383345	162449208	40820476	516207494	57266809	3788615
**CV%**	**56.7**	**49.5**	**50.2**	**42.2**	**59.3**	**38.0**

Table S2. **Summary descriptive statistics**. Data are color-coded as follows: nuclear (blue), mitochondrial (pink), and cytosolic (green). For each fraction, the minimum and maximum values are provided, along with the range, which corresponds to the difference between the maximum and minimum values, mean, standard deviation (Std. Deviation), standard error of the mean (Std. Error of Mean), and coefficient of variation (CV%) are provided. The CV was calculated as the standard deviation divided by the mean. The left column corresponds to the cellular approach, whereas the right column corresponds to the tissue-based approach.

11.5 Table S3. **Summary statistics for technical replicates.**

	Prelamin-A/C			
	Cytosol	*Mitochondrial*	*Nuclear*	*Proteome total*
Minimum	15570633	125545098	1643382588	558625364
Maximum	21797757	143620540	1813365118	645528504
Range	6227124	18075442	169982530	86903140
Mean	18611594	136988883	1756060798	602076934
Std. Deviation	3116100	9952278	97586966	43451570
Std. Error of Mean	1799081	5745950	56341861	25086776
**CV%**	**16.7**	**7.3**	**5.6**	**7.2**

	Nucleophosmin			
	Cytosol	*Mitochondrial*	*Nuclear*	*Proteome total*
Minimum	117519140	35176298	454356032	57155878
Maximum	140233532	54312044	588447488	100047730
Range	22714392	19135746	134091456	42891852
Mean	125090604	41554880	499053184	78601804
Std. Deviation	13114160	11048028	77417738	21445926
Std. Error of Mean	7571464	6378582	44697152	12381811
**CV%**	**10.5**	**26.6**	**15.5**	**27.3**

	ATPase			
	Cytosol	Mitochondrial	Nuclear	Proteome total
Minimum	56990183	1376143488	94748506	1489841920
Maximum	154924724	4256963136	331445928	2010290048
Range	97934541	2880819648	236697422	520448128
Mean	95351972	2401345621	180704164	1750065984
Std. Deviation	52299353	1609960478	130974605	260224064
Std. Error of Mean	30195045	929511115	75618223	150240433
CV%	**54.8**	**67.0**	**72.5**	**14.9**

	CSP1			
	Cytosol	Mitochondrial	Nuclear	Proteome total
Minimum	645501568	1282465536	59075372	811405760
Maximum	649594176	1702267904	70417224	1463506176
Range	4092608	419802368	11341852	652100416
Mean	647547872	1492366720	64746298	1137455968
Std. Deviation	2046304	209901184	5670926	326050208
Std. Error of Mean	1181434	121186505	3274111	188245175
CV%	**0.3**	**14.1**	**8.8**	**28.7**

	HS90B			
	Cytosol	Mitochondrial	Nuclear	Proteome total
Minimum	1788563768	246534830	131315359	846397907
Maximum	1868264580	312871016	147081860	1080717004
Range	79700812	66336186	15766501	234319097
Mean	1825588356	289989590	138518984	963557456
Std. Deviation	40149852	37650617	7970653	117159549
Std. Error of Mean	23180528	21737594	4601859	67642097
CV%	**2.2**	**13**	**5.8**	**12.2**

Table S3. **Descriptive statistical parameters corresponding to technical replicates.** Summary statistics, including minimum, maximum, range, mean, standard deviation (Std. Deviation), standard error of the mean (Std. Error of Mean), and coefficient of variation (CV%), are provided for each protein across subcellular fractions. Data are color-coded according to subcellular localization: nuclear (blue) Prelaminin A/C and Nucleophosmin, mitochondrial (pink) CPS1 and ATPase, cytosolic (green) HS90B.
